# Automated quality control analysis for American College of Radiology (ACR) digital mammography (DM) phantom images

**DOI:** 10.1002/acm2.14548

**Published:** 2024-10-09

**Authors:** Zeyad Alawaji, Seyedamir Tavakoli Taba, Lucy Cartwright, William Rae

**Affiliations:** ^1^ Discipline of Medical Imaging Science Faculty of Medicine and Health The University of Sydney Sydney New South Wales Australia; ^2^ Department of Radiologic Technology College of Applied Medical Sciences Qassim University Buraydah Saudi Arabia; ^3^ Medical Physics Department Western Sydney Local Health District Westmead New South Wales Australia; ^4^ Medical Imaging Department Prince of Wales Hospital Randwick New South Wales Australia

**Keywords:** image analysis, mammography, phantom, quality control, software

## Abstract

**Purpose:**

To develop and validate an automated software analysis method for mammography image quality assessment of the American College of Radiology (ACR) digital mammography (DM) phantom images.

**Methods:**

Twenty‐seven DICOM images were acquired using Fuji mammography systems. All images were evaluated by three expert medical physicists using the Royal Australian and New Zealand College of Radiologists (RANZCR) mammography quality control guideline. To enhance the robustness and sensitivity assessment of our algorithm, an additional set of 12 images from a Hologic mammography system was included to test various phantom positional adjustments. The software automatically chose multiple regions of interest (ROIs) for analysis. A template matching method was primarily used for image analysis, followed by an additional method that locates and scores each target object (speck groups, fibers, and masses).

**Results:**

The software performance shows a good to excellent agreement with the average scoring of observers (intraclass correlation coefficient [ICC] of 0.75, 0.79, 0.82 for speck groups, fibers, and masses, respectively). No significant differences were found in the scoring of target objects between human observers and the software. Both methods achieved scores meeting the pass criteria for speck groups and masses. Expert observers allocated lower scores to fiber objects, with diameters less than 0.61 mm, when compared to the software. The software was able to accurately score objects when the phantom position changed by up to 25 mm laterally, up to 5 degrees rotation, and overhanging the chest wall edge by up to 15 mm.

**Conclusions:**

Automated software analysis is a feasible method that may help improve the consistency and reproducibility of mammography image quality assessment with reduced reliance on human interaction and processing time.

## 1 INTRODUCTION

Mammography is the preferred imaging modality in breast screening programs, as it has been shown to considerably decrease breast cancer mortality, by enabling early diagnosis of breast cancers.^1^ Achieving an optimal balance between image quality and radiation dose is fundamental to ensuring accurate interpretation of mammograms whilst also minimizing the radiation burden to the screened population of women. Several quality control (QC) guidelines in mammography have been developed and implemented nationally and internationally to ensure optimal mammography performance in clinical settings.^2^, ^3^, ^4^

A quality assurance program usually consists of numerous QC tests carried out according to a standard schedule by appropriately trained staff. Some of these tests are performed by medical imaging technologists/radiographers, while others should be completed by qualified medical physicists.^5^, ^6^ Most tests used in QC assessments require substantial user interaction, which can be time‐consuming and susceptible to inter‐ and intra‐observer variation.^2^, ^7^ One critical component of QC testing is mammographic image quality assessment. This is usually performed weekly. Typically, the assessment is conducted by a technologist who acquires a standard image of a recognized accreditation phantom. These phantoms contain objects that mimic breast pathologies, such as masses, fibers, and microcalcifications. These objects usually have a range of sizes and contrasts. The subjectively evaluated score assigned to each object is determined by the clarity with which it can be seen. Each object type's entire score is compared with the minimum acceptable score of image quality.^8^, ^9^, ^10^

Considering humans’ cognitive and visual limitations, several studies have been done to use automated digital image analysis for assessment of the accreditation phantom images.^2^, ^4^, ^7^, ^11^, ^12^, ^13^ A variety of image analysis methods have been described in the literature which aim to quantitatively assess the images of the embedded objects of the accreditation phantoms. These methods include the use of discrete wavelet transforms (DWT),^2^, ^8^ template matching,^8^, ^14^, ^15^, ^16^, ^17^, ^18^, ^19^ Mahalanobis distance classifiers,^17^ and Gray‐Level Co‐Occurrence Matrix (GLCM) analysis.^20^ Since the resulting scores are more consistent and reproducible compared to human assessments, computerized analyses can detect small variations in the images' quality by tracking and analyzing the recorded scores over the long term.^2^, ^11^

It is pertinent to note that the most frequently recommended phantoms in Europe and the UK were CDMAM and Leeds test objects (e.g., TORMAM, TORMAX). There exists well‐established automated analysis software for these phantom images supplied by the manufacturer.^3^ However, the ACR phantom which is standard in many countries such as the US, Canada, and Australia,^3^ does not yet have standard analysis software. In this study, we aimed to develop and validate an automated method for the analysis of ACR full‐field digital mammography (DM) phantom images acquired on mammography units. The software was programmed to perform image quality assessment according to the Royal Australian and New Zealand College of Radiologists (RANZCR) guideline,^21^ which is generally in alignment with the ACR guideline.^22^ This could be implemented in current practice to allow reproducible image quality evaluation and thereby reduce the time needed for assessment while avoiding variations due to subjective human observer evaluation.

## 2 MATERIALS AND METHODS

### 2.1 Phantom description

**FIGURE 1:**
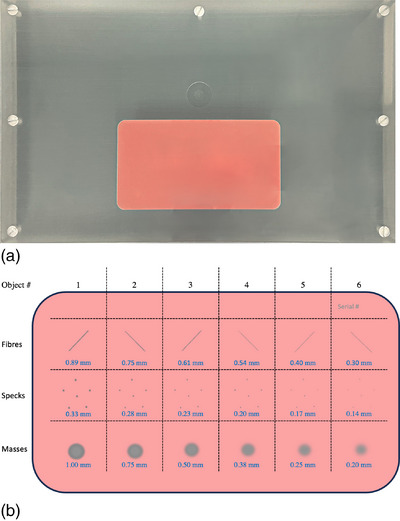
(a) an example of the ACR DM phantom. (b) Schematic view of wax block showing the included structures consist of six fibers, six specks groups, and six masses, along with their respective sizes.

In this study, the ACR DM phantom (Model 086) was utilized as the DM accreditation phantom for QC.^22^, ^23^ This phantom was developed following wide acceptance of direct DM. It has finer gradations and smaller objects compared to the former ACR phantom to be more sensitive to changes in a DM system. The physical dimension of this polymethyl methacrylate (PMMA) phantom is 31 × 19 cm with a thickness of 4.1 cm, which covers most of the detector area and simulates the x‐ray attenuation of a compressed breast of average glandular/ and adipose composition. The phantom contains two main components with known size and location: first, the signal difference‐to‐noise ratio (SDNR) cavity, and second, a wax block that contains six test objects for each of three different object types. These object types include fibers (diameters range from 0.33 mm up to 0.80 mm), masses (thicknesses range from 0.20 mm up to 1.00 mm) and groups of six microcalcifications (sizes ranging from 0.14 mm up to 0.33 mm) that mimic breast pathologies and vary in size and contrast. Figure 1 shows the ACR DM phantom image.

### 2.2 Mammography systems and image acquisition

**FIGURE 2:**
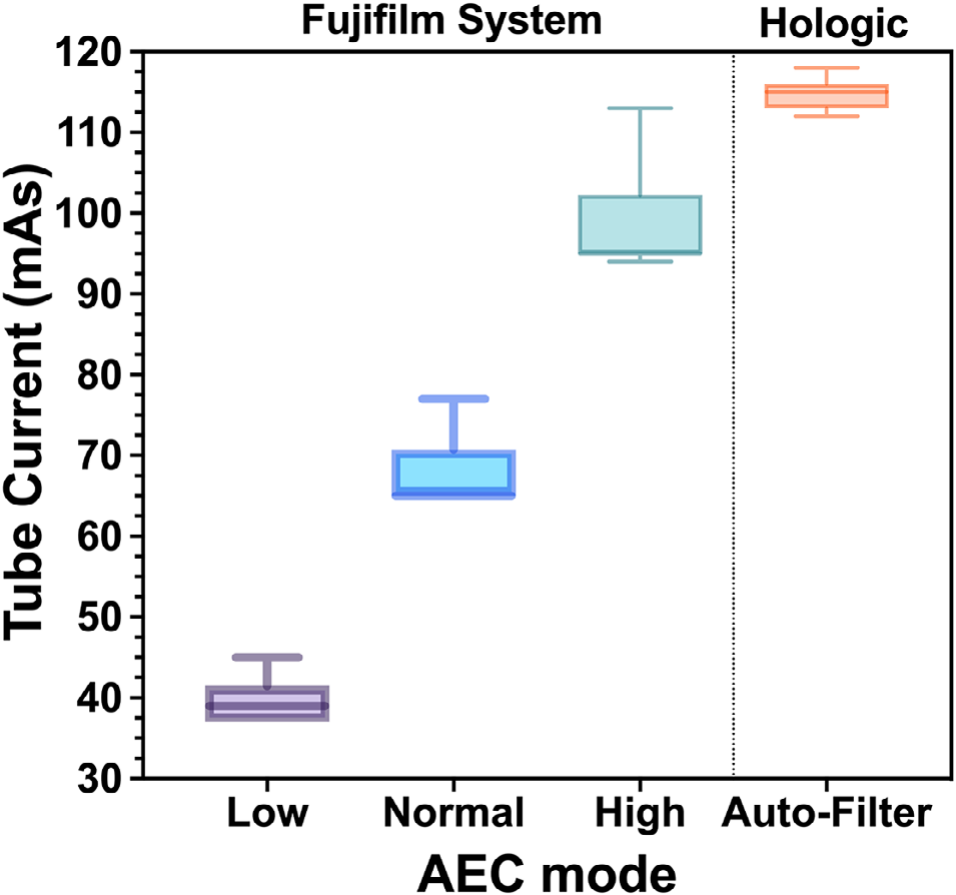
The acquisition factors for the ACR DM phantom images used in this study for Fujifilm and Hologic Systems. Note: kVp ranges between 28 and 29 kVp.

Two different DM system vendors were utilized in this study. Initially, twenty‐seven processed “for presentation” images of the ACR DM phantom were used to benchmark the algorithm against human scoring. These images were collected at various times over the course of a year from BreastScreen accredited service providers in New South Wales, Australia. These images were exported in Digital and Imaging Communications in Medicine (DICOM) format from two FUJIFILM digital mammography systems (Amulet Innovality, FUJIFILM, Japan), which were running under different software versions. It is evident that the image processing algorithms varied between the two systems, resulting in significant variations in pixel values. The systems utilize a direct‐conversion flat panel detector made of Amorphous Selenium (a‐Se), featuring a pixel pitch of 50 µm, Table 1 presents the main system specifications and acquisition technique. To assess algorithm performance, we deliberately acquired phantom images that were expected to have different scores compared to the standard images. The Automatic Exposure Control (AEC) mode, commonly employed in clinical settings, was used in three different modes: low, normal, and high. The tube voltage was constant, with variable mAs, and used Tungsten (W)/Rhodium (Rh) as the target/filter combination, Figure 2 shows the distribution of the tube current (mAs) across different AEC modes. To further evaluate the robustness of our algorithm, we tested its applicability and sensitivity across different imaging conditions using a Selenia Dimensions (Hologic Inc., USA) mammography system (Table 1). We deliberately acquired twelve ACR DM phantom DICOM images “for presentation” under typical clinical AEC exposure parameters (Auto‐Filter AEC mode) but varied the phantom positioning to simulate potential operator positioning variations that might occur in a clinical setting. The variations included lateral movements of the phantom by 15 and 25 mm to both the left and right, rotations of approximately 2.5 and 5 degrees to both the left and right, overhanging the chest wall edge by 25 and 15 mm, and underhanging by 25 and 15 mm. These tests were designed to assess the algorithm's sensitivity to different extremes in positioning and its ability to consistently reproduce results under varied imaging conditions. All systems used in this study have regular QC tests to meet the performance requirements set by the RANZCR guidelines for QC Testing for DM.^23^

**TABLE 1:** The main specifications of the systems and the acquisition technique.

Manufacturer	Fujifilm	Hologic
Model	Amulet Innovality	Selenia Dimensions
X‐ray tube target/filter	W/Rh	W/Rh
Detector technology	Full‐field direct amorphous Selenium (a‐Se)	Direct amorphous Selenium (a‐Se)
Detector size (cm)	24 × 30	24 × 29
Pixel pitch (µm)	50	70
Grid	In	In
AEC mode	Low/Normal/High	Normal
Source to image receptor distance: SID (mm)	650	700
Software versions	FDR‐3000AWS Mainsoft V9.0 and V7.0.0017	AWS:1.10.0.412

### 2.3 Human observer scoring

The 27 phantom images obtained from FUJIFILM (Amulet Innovality) systems were evaluated by three expert medical physicists who were qualified as observers, under clinical viewing conditions. The images were scored according to the RANZCR mammography QC guideline, which involves counting the most visible and largest object in each object group (i.e., masses, speck groups, fibers) until a score of zero is reached for a given object.^23^ The observers assigned a score of [0, 0.5, 1] to each test object based on whether it was not visible, partially visible, or completely visible, respectively. They then recorded a total summation score for each object group. To pass the image quality assessment according to the RANZCR criteria, the scores for the fibers, masses, and specks groups should be 4, 3, and 3, respectively.^23^ However, in our study, the observers were instructed to continue scoring even if the accepted score threshold was reached, to evaluate the algorithm's performance against human observers. The observers were not informed during the scoring about the system or the conditions under which the image was acquired.

### 2.4 Image analysis approach

The ACR DM phantom analysis software was developed using MATLAB (The MathWorks Inc., Natick, Massachusetts, USA), to fully automate the extraction and analysis of several regions of interest (ROIs) from the phantom images. Initially, the relevant attributes of DICOM metadata were extracted for each image since the orientation of the image and the pixel spacing information were essential to getting the correct orientation and measurement. Morphological processing was applied to extract the wax block based on the known size of the phantom structure. This identification is aided by the pixel size information from the DICOM tag. Subsequently, the wax block was divided into 18 ROIs to isolate each test object (fibers, speck groups, masses), allowing the software analysis and scoring of each subregion separately, as shown in Figure 1.

**FIGURE 3:**
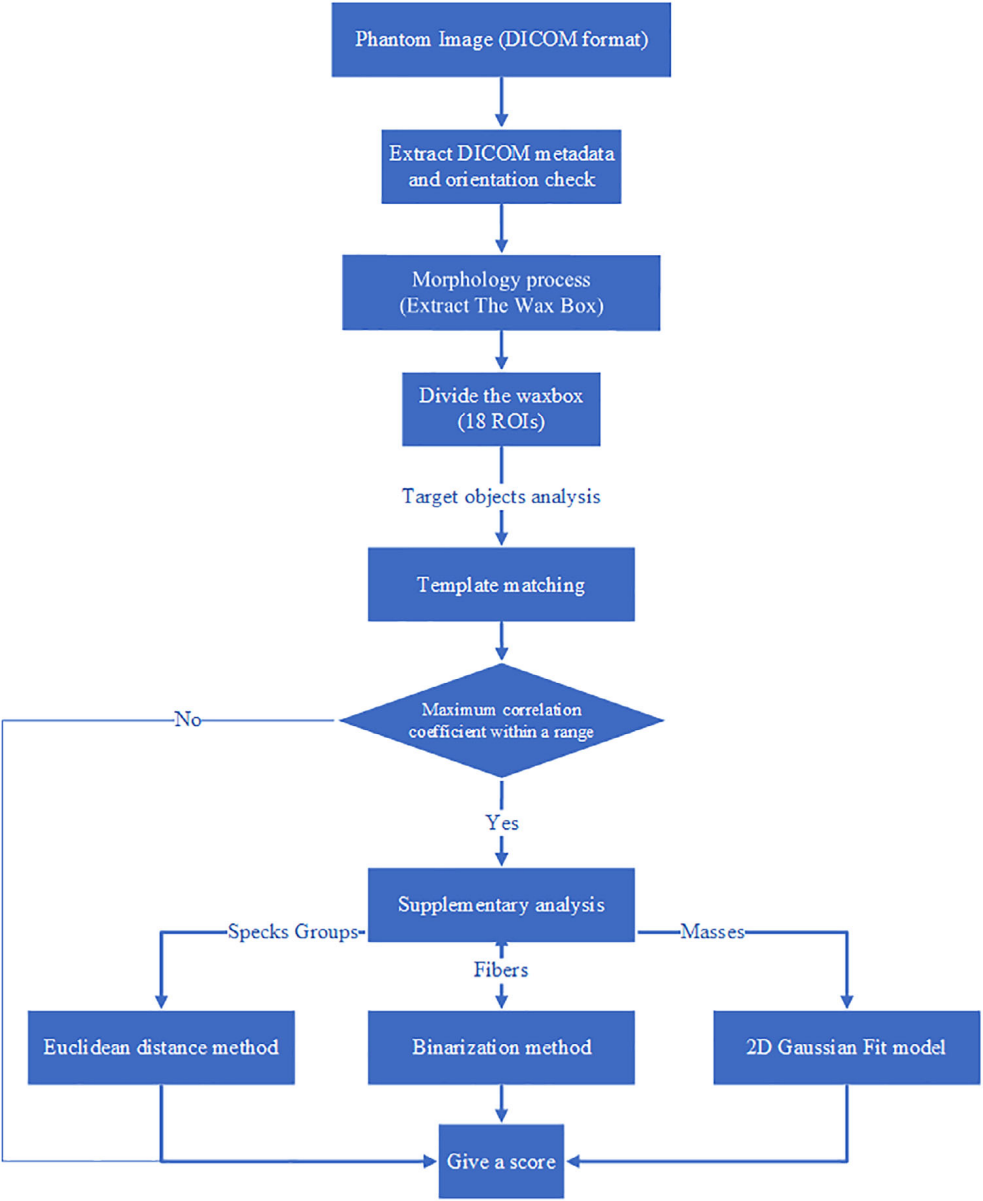
Flowchart of the automated analysis software workflow.

In the initial stage of algorithm development, target object detection was performed without any denoising process. However, due to the prevalence of noise, the algorithm failed to correctly identify the smallest specks, resulting in a high rate of false positives. This finding agrees with another study,^2^ which noted that the smallest speck groups often get obscured by noise. To reduce the noise, the 18 ROIs were exclusively smoothed using a Gaussian filter, which improves the detection of the objects by increasing the signal‐to‐noise ratio (SNR) and minimizing misinterpretation. Otherwise, all analyses were performed on the original unaltered images without any modifications. Figure 3 shows a flowchart of the automated analysis software.

### 2.5 Fibers, speck groups, and masses analysis

The fibers, speck groups, and masses were analyzed by applying two different analyses on each object to reduce false positive detection, before considering the object detected for scoring.

### 2.6 Template matching method

Firstly, for each object, the template matching technique was applied, whereby a template image is shifted across the sub‐image being analyzed pixel by pixel to calculate the correlation coefficient similarity score. This was achieved by using the 2D normalized cross‐correlation function (normxcorr2) in MATLAB, which has been described previously by Gonzales et al.^24^ and is given by Equation (1)^25^:

(1) $$\gamma \left(u,v\right)=\frac{\sum_{x,y}\left[f\left(x,y\right)-{\bar{f}}_{u,v}\right]\left[t\left(x-u,v-v\right)-\bar{t}\right]}{{\left\{\sum_{x,y}{\left[f\left(x,y\right)-{\bar{f}}_{u,v}\right]}^{2}\sum_{x,y}{\left[t\left(x-u,v-v\right)-\bar{t}\right]}^{2}\right\}}^{0.5}}$$

where ƒ(x,y) and *t* represent the sub‐image and the template, respectively, and $\bar{f}$ and $\bar{t}$ are the mean value of the image and the template, respectively. The result would be the position of maximum correlation and similarity.

**FIGURE 4:**
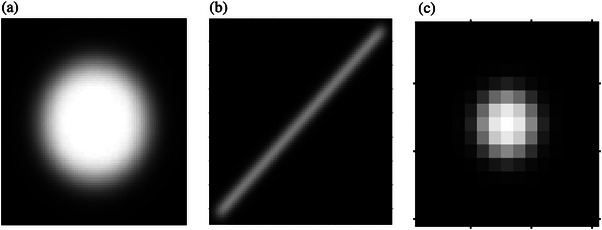
Template images for (a) a mass (80 × 80 pixels), (b) a fiber (80 × 80 pixels), and (c) an individual speck (15 × 15 pixels). Note the pixels seen are due to differing zoom factors on these zoomed images.

Binary template images were created automatically, considering the shape, orientation, and similar physical size of each targeted test object based on the phantom and the mammography system specifications. An example of the templates is shown in Figure 4.

If the maximum correlation coefficient fell within the set range of values determined by the average score of the observers for each object, the software proceeded to the supplementary method. Otherwise, the object received a score of zero, the scoring of the objects group stopped, and the total score of the object group was tallied.

### 2.7 Supplementary analysis methods

**FIGURE 5:**
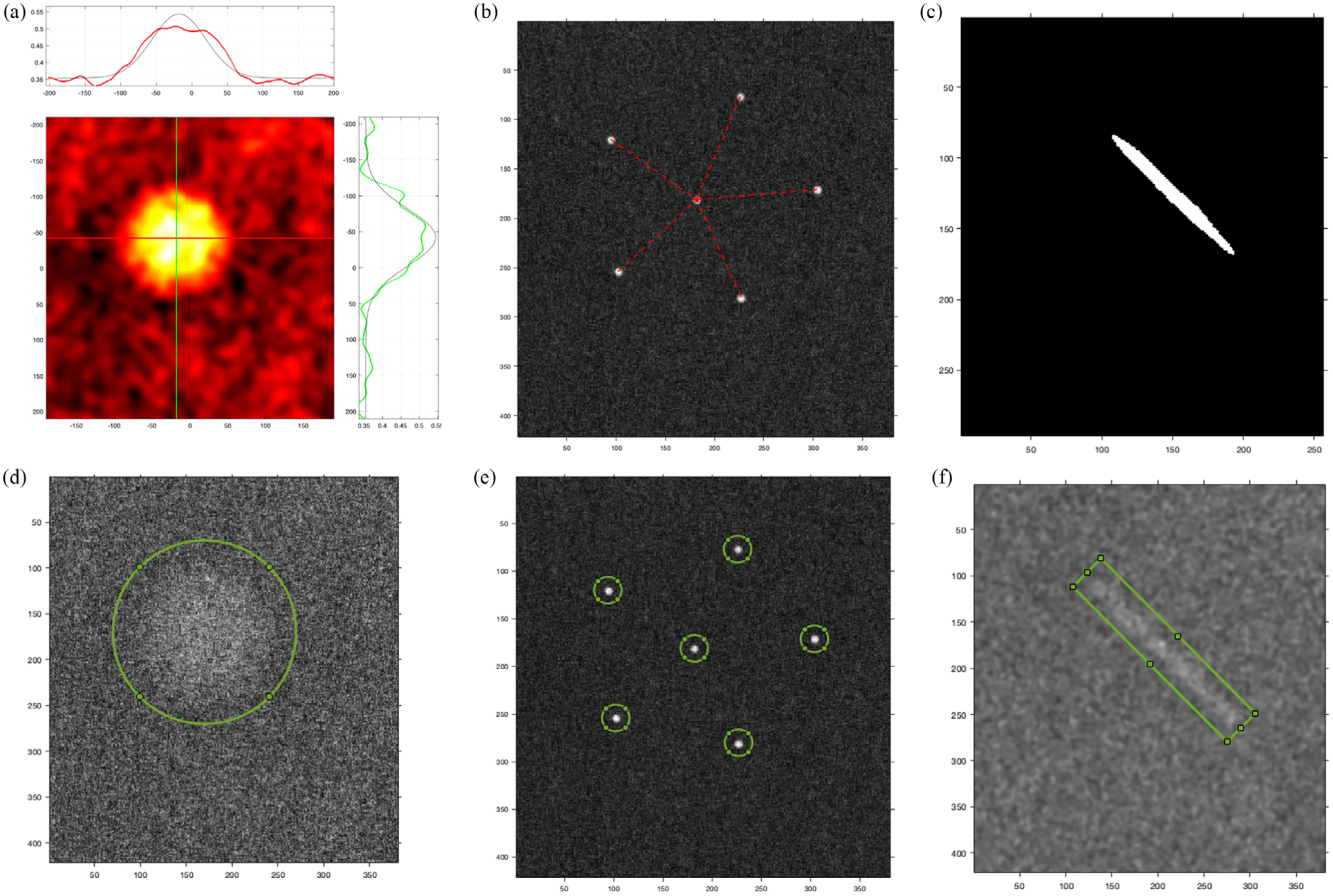
An illustration of supplementary methods used for each test object (a) Gaussian fit model for masses. (b) Euclidean distances for speck groups. (c) Image binarization for fibers. (d–f) ROIs for each detected test object. Note: Some images have been resized or zoomed for clarity.

For masses, the 2D Gaussian function located in the MATLAB file exchange was applied.^26^ This function uses the least square curve fitting approach to estimate the centroid and the width of a mass in the ROI by fitting the intensity profile in the x and y directions. An example of the mass result plot is shown in Figure 5a.

To obtain the best fitting curve, initial guess parameters must be close to the final results. Therefore, multiple initial guesses were set for each mass size. The 2D Gaussian fitting was found using the formula:

(2) $$f\left(x,y\right)=A*\exp\left(-\frac{{\left(x-x_{0}\right)}^{2}}{2\sigma_{0}^{2}}+\frac{{\left(y-y_{0}\right)}^{2}}{2\sigma_{0}^{2}}\right)+Bkg$$

where (*A*) is the amplitude (maximum intensity), *x*_0_, *y*_0_, the center coordinates of the mass, *σ*_0_, represents the width (standard deviations) of the mass on two axes, and the constant variable, *Bkg*, refers to the background.

After the Gaussian function is applied, the software extracts the centroid coordinate of the mass fit and the FWHM to add it with the template matching results and determine the visibility of the object (Figure 5).

For the speck groups, as the approximate distance between each speck within a group is known, the 2D Euclidean distance equation was applied, which calculates the distance between the speck at the center of the sub‐image to other specks within a group.

(3) $$\text{Euclidean distance}=\surd \left[{\left(x_{i}-x_{1}\right)}^{2}+{\left(y_{i}-y_{1}\right)}^{2}\right]$$

where (*x*_1_,*y*_1_) is the speck at the center, and (*x*_i_,*y*_i_) is the coordinate of speck, *i*, detected by the template matching technique, as shown in Figure 5b.

The specks within a range of acceptable distance were determined as a true positive detection of the template matching. Then, the number of detections was counted, and a score was assigned based on the number of specks detected in each ROI. If ≥ 4 specks were found, a score of 1 was assigned; findings of ≥2 but ≤ 3 was scored as 0.5, and less than 2 was considered not detected (Figure 5).

For fibers, the cross‐correlation image was segmented as shown in Figure 5c to extract the orientation feature of the fiber using the binary large object (BLOB) detection technique. If the fiber orientation was within the ranges of 45° ± 4° (for fibers 1, 3, and 5) or 135° ± 4° (for fibers 2, 4, and 6), it was considered for scoring (Figure 5).

### 2.8 Statistical analysis

The intraclass correlation coefficient (ICC) was obtained to assess the inter‐rater reliability between the average human observers scores and the software scores using (a two‐way mixed effect model with consistency agreement).^27^, ^28^ ICC reliability value threshold of >0.60 indicates good to excellent reliability according to Cicchetti's (2001) recommendations, and a threshold >0.50 indicates moderate to good reliability according to Koo and Li's (2016) guidelines. The nonparametric pairwise comparison (Wilcoxon signed‐rank test)^29^ was used to determine the significant difference between the observer scores and software scores for each test object. The scoring differences across different dose levels were assessed using the Kruskal–Wallis test with Bonferroni correction, and the Mann–Whitney test was used to test if there were any differences between the two software versions in terms of scoring. In addition, the scoring agreement between both methods was assessed using Bland–Altman plots. SPSS statistical software (SPSS Inc, Chicago, IL, USA) and GraphPad Prism version 9.0 (GraphPad Software, La Jolla, CA, USA) were used to conduct the statistical analysis at a 5% significance level.

## 3 RESULTS

### 3.1 Software assessment in comparison with human scoring

In a dataset containing 27 images, target objects including fibers, specks, and masses were accurately located using automated software. Out of the total number of images scored (27 × 3), 11 images failed to meet the minimum image quality score of 4 for fibers (*n* = 10), 3 for speck groups (*n* = 0), and 3 for masses (*n* = 1). These were most often due to the fiber target objects, with 37%, 18.5%, 7.4%, and 7.4% failures reported by observer 1, observer 2, observer 3, and the software, respectively. In contrast, only one image failed in the mass evaluation conducted by observer 2, and there were no failures in the speck groups. It is worth noting that all images passed the image quality assessment based on the ACR guidelines score criteria,^22^ which require a minimum score of 2, 2, and 3 for the fibers, masses, and speck groups, respectively. The Mann–Whitney test showed no statistically significant differences in image scoring between the two software versions of the mammography system (*p* > 0.05), indicating that the pixel value variations did not significantly impact the scoring.

The average pairwise differences between the software and average human observers' scores for detecting fiber objects on the FUJIFILM mammography systems (Amulet Innovality), were 0.61, 0.32, −0.23 for low, normal, and high dose modes, respectively. This indicates that the software was more sensitive in detecting fiber objects at low and normal dose modes compared to human observers. Based on the pairwise comparison, there was no statistically significant difference between the software and human observers (*p* = 0.1). ICC between the average scores of the human observers and software indicates good to excellent reliability (0.79).

For specks, the average pairwise differences between the software and average human observers' scores on the FUJIFILM system were −0.56, 0.17, and 0.54 for low, normal, and high‐dose modes, respectively, indicating that the software was less sensitive under low dose mode when assessing high contrast objects. There was no significant difference between the software and human observers in the paired comparison (*p* = 0.9). The ICC between the average scores of the human observers and the software indicates good to excellent reliability (0.75).

The average pairwise differences between the software and average human observers' scores in detecting mass objects on the FUJIFILM system were 0.28, 0.02, and −0.10 for low, normal, and high dose modes, respectively. In the paired comparison analysis, there were no significant differences between the software and human observers (*p* < 0.8). Excellent reliability was demonstrated by the ICC between the average scores of the human observers and the software score (0.82).

**FIGURE 6:**
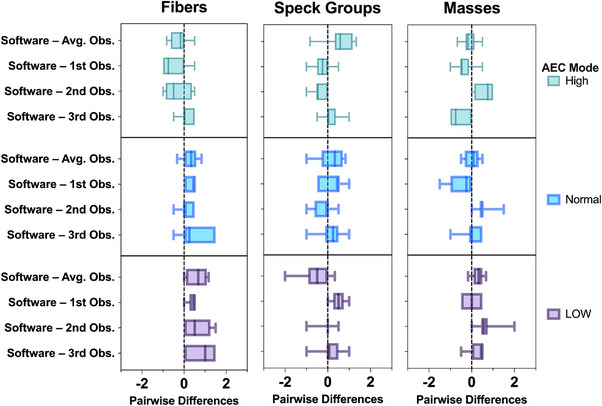
Average pairwise differences in scoring between observers and software across different dose level modes for each target object in the phantom.

Figure 6 presents the average pairwise differences in scoring between the software and human observers. Generally, the software tended to score slightly higher than the observers for low‐contrast objects in low and normal AEC modes. In contrast, observers tend to score slightly higher in the high‐dose mode.

As outlined in Table 2, the Kruskal–Wallis test showed statistically significant differences (*p* < 0.05) in image scoring between the low and high dose levels for all target objects. Additionally, the speck groups demonstrated significant differences between low and normal doses, as well as the masses between normal and high doses.

**TABLE 2:** *P*‐values for comparisons of image scoring by software and average observers at different dose level modes.

Target objects	Comparison dose level modes	Software *p*‐value	Observers *p*‐value
Fibers	Low vs. normal	0.246	0.082
	Low vs. high	**0.002**	**<0.001**
	Normal vs. high	0.241	0.137
Specks	Low vs. normal	0.016	**0.013**
	Low vs. high	**0.001**	**<0.001**
	Normal vs. high	1.000	0.162
Masses	Low vs. normal	0.872	0.200
	Low vs. high	**<0.001**	**<0.001**
	Normal vs. high	**0.013**	**0.049**

The bold values represent the statistically significant results at a specified level *p* < 0.05.

**FIGURE 7:**
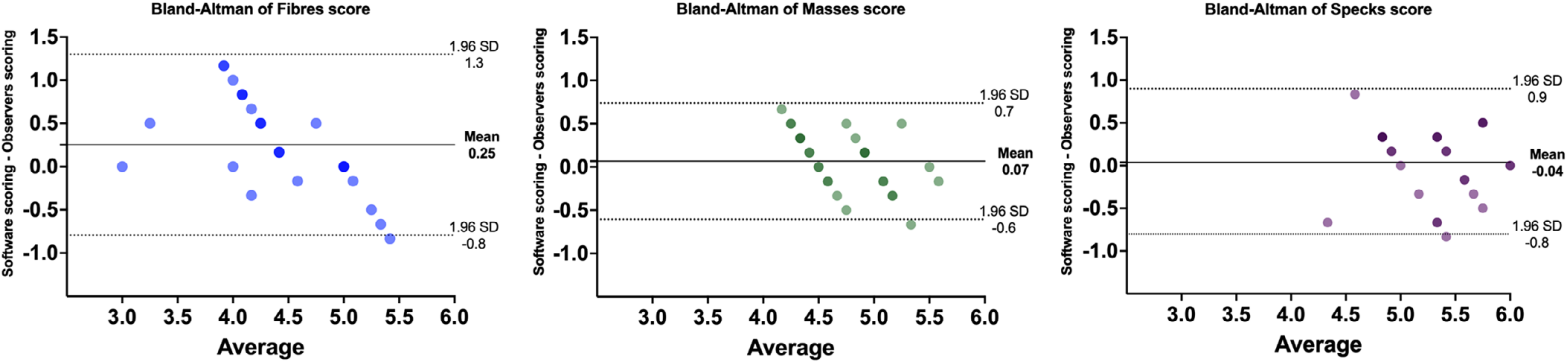
Bland–Altman plots showing the difference in scoring between the software and the human observers. The solid horizontal line is the mean difference between the software and the average of the human observers scores, and the two dashed lines represent the upper/lower limit of agreement within ± 1.96 SD.

Figure 7 displays the Bland–Altman plots that indicate the level of agreement between the software and the average of the human observers in scoring the 27 images. The mean difference between the two methods was 0.25, −0.04, and 0.07 for fibers, speck groups, and masses, respectively. The graph illustrates the variability in the scoring, particularly for fiber scores, whereas the highest agreement was observed in the speck groups and masses scores. However, overall, the scoring of all target objects showed good agreement between the software and human observers.

### 3.2 Software scoring sensitivity to phantom positioning

By testing the algorithm through twelve different positions of the phantom, we were able to assess its robustness and sensitivity to positioning errors that may be encountered in clinical practice. The algorithm produced scores that met minimum acceptable image quality standards for most positioning error scenarios, including lateral motions, rotational adjustments, underhanging, and overhanging by 15 mm of the chest wall edge. However, the algorithm failed to determine a score when the phantom extended beyond the margin of the chest wall by 25 mm. The algorithm's failure to appropriately assess the images was caused by the extreme position, which led to a partial cut‐off of the wax box. The images were scored by a medical physicist, and the average difference between the software and human observer scores were within 0.5 for fibers, speck groups, and masses.

Table 3 provides an overview of the scoring results for different positional adjustments. All target objects were accurately identified and scored, where all images passed the scoring criteria of the RANZCR mammography QC guideline. The algorithm demonstrates its resilience to these operational variations except for the extreme 25 mm case where the phantom overhung the chest wall side of the patient support.

**TABLE 3:** Scoring results of target objects by the software across various phantom positional adjustments.

Phantom positioning	Fibers	Speck groups	Masses
Lateral movement to the left by 15 mm	5	5.5	6
Lateral movement to the left by 25 mm	5	5.5	5
Lateral movement to the Right by 15 mm	5	5	6
Lateral movement to the Right by 25 mm	5	6	5
Rotation of approximately 2.5° to the right	5	6	6
Rotation of approximately 2.5° to the left	4	5.5	5
Rotation of approximately 5° to the right	5	5.5	6
Rotation of approximately 5° to the left	4	6	6
Underhanging the chest wall edge by 15 mm	5	5.5	5
Underhanging the chest wall edge by 25 mm	5	5	5
Overhanging the chest wall edge by 15 mm	4	5.5	5
Overhanging the chest wall edge by 25 mm	Fail	Fail	Fail

## 4 DISCUSSION

To improve the consistency and efficient analysis and scoring of ACR DM phantom images, this study describes the development and validation of in‐house software for analyzing and scoring these images. The software provides higher consistency and reproducibility than human observers, thus reducing the time and variation caused by subjective human assessment. Our in‐house software's performance was evaluated by comparing its output with human image quality assessment, and the results show excellent agreement between the two methods.

Besides the promising performance of this automated method for the analysis of ACR DM phantom images acquired on mammography units, the software has the general advantage of consistency, which significantly reduces the potential impact of external factors that could bias human observers and lead to inconsistent results. These factors may include the lighting conditions, image display monitors and observer expertise, and fatigue. The software operates in an automated way with minimal human interaction, which could potentially improve the accuracy and consistency of ACR DM phantom image analysis.

The results of the study show that the software is effective in scoring ACR DM phantom images at different dose level modes. Also, it achieved good to excellent reliability, and the pairwise comparison shows no significant differences compared to human observers' scoring of each target object. The correlation coefficient ranged from 0.75 to 0.82, indicating a strong agreement between the software and observers. The higher agreement observed in speck groups and masses scoring between the software and the observers may be attributed to the reduced variation in scoring among observers when assessing high‐contrast objects and well‐defined structures, in comparison to the evaluation of low contrast fiber objects where the scoring tends to be less consistent among different observers, specifically in low dose mode. The variations in the observers' scores might be due to several reasons, including human subjective scoring. It is noteworthy that the observers tend to assign lower scores to the fiber objects, falling below the passing criteria outlined in the RANZCR guideline^23^ compared to the software. This finding suggests that the software demonstrates reliable performance in scoring objects with low contrast. While the software shows promising performance, further investigation is required to determine the optimal balance between the software sensitivity and human perception in clinical practice. This may include tuning the software to provide results that are more consistent with human observers.

The findings show that the software aligns perfectly with observer assessments in identifying failed images. Nevertheless, all images satisfied the quality image assessment based on the ACR guidelines score criteria,^22^ requiring scores of 2, 2, and 3 for the fibers, masses, and speck groups, respectively.

The software's robustness was assessed using mammography systems from two different vendors and multiple software versions from a single vendor. This approach allowed us to evaluate the software's ability to analyze images across two system designs and imaging conditions, thereby broadening the generalizability of our results. This diversity in image processing confirmed that our software can effectively analyze a range of processed images. The software consistently exhibited robust performance, even under extreme variations in phantom positioning, such as overhanging and underhanging at the chest wall edge. Notably, an overhang of 25 mm at the chest wall edge led to image detail truncation, particularly affecting the wax box in the images. This observation highlights the critical importance of careful phantom positioning in clinical settings, and quick review by a human observer to ensure accuracy and reliability.

While previous studies have performed automated assessments of images obtained using the previous version of the ACR phantom, as well as other accredited phantoms,^2^, ^12^, ^14^, ^30^ none have reported or tested automated software on ACR DM phantoms, which is considered to be more sensitive for DM compared to the previous version. Also, we have chosen the ACR DM phantom due to its being widely accepted by regulatory bodies and it is commonly adopted. This supports standardization and comparability of QC metrics across different institutions. Furthermore, in this study, we deliberately avoided extensive post‐processing of the original images to maintain their clinically relevance status, which will finally be evaluated by human observers. Similar to our approach, several studies have utilized the template matching method to determine the visibility of the objects on the previous version of the ACR phantom.^14^, ^16^ However, we added a supplementary method for each object to minimize the false‐positive detection of template matching. Our findings cannot be compared with the results of previous studies due to the differences in the phantom structure; however, it is aligned with previous research, which indicates that low‐contrast objects are the most challenging for human observers due to the subjective evaluation process and environmental factors, such as lighting conditions.^2^

A limitation of this study was our inability to fully assess our algorithm's performance since we did not include extremely low‐quality images in our study. It should be noted that the phantom images used in our study were obtained using well‐maintained mammography systems. To evaluate our software's robustness, we deliberately included images acquired with atypical parameters, utilizing three distinct modes of AEC: low, normal, and high on the FUJIFILM mammography systems (Amulet Innovality). Although the quality of these images failed to meet the stricter standards of RANZCR scoring criteria in Australia, they did comply with the U.S. ACR standards. This hindered our ability to fully evaluate our algorithm's performance on extremely poor‐quality images.

Our work aimed to validate the software analysis method using the ACR DM phantom images obtained under clinical operation conditions, without any extensive enhancements. The findings of this study indicate that the software we developed demonstrated comparable performance to human observers. Additionally, it shows improved consistency and reproducibility, making it suitable for long‐term QC tracking. Nonetheless, this study serves as a proof of concept, and further research is necessary to validate the software's performance using a larger dataset of images. This should incorporate images from different mammography manufacturers that use diverse post‐processing algorithms or detector technologies. This will ensure reliable and consistent results across multiple systems.

## 5 CONCLUSIONS

The developed software effectively automated and standardized the current subjective image QC assessment. This automation enhances the efficiency of conducting image quality testing more frequently, free from the impact of subjective assessments. An automated quality assessment software would provide a valuable tool for assessing image quality in a consistent and standardized manner, which may enhance the accuracy and reliability of mammography QC.

## AUTHOR CONTRIBUTIONS

Z. Alawaji, S. Taba, and W. Rae contributed to the conception and design of the study. Z. Alawaji developed and validated the software, performed statistical analyses, curated the data, and drafted the original manuscript. Z. Alawaji and L. Cartwright provided resources. All authors contributed to the interpretation of the data, reviewed and edited the manuscript, and approved the final version for submission.

## CONFLICT OF INTEREST STATEMENT

The authors declare no conflicts of interest.

## Data Availability

The data that support the findings of this study are available from the corresponding author upon reasonable request.
